# Dressing Tool Condition Monitoring through Impedance-Based Sensors: Part 1—PZT Diaphragm Transducer Response and EMI Sensing Technique

**DOI:** 10.3390/s18124455

**Published:** 2018-12-16

**Authors:** Pedro Junior, Doriana M. D’Addona, Paulo R. Aguiar, Roberto Teti

**Affiliations:** 1 Faculdade de Engenharia, Department of Electrical Engineering, UNESP-University Estadual Paulista, Bauru, Av. Eng. Luiz Edmundo C. Coube 14-01, Bauru–SP 17033-360, Brazil; pedrojunior5@aedu.com (P.J.); aguiarpr@feb.unesp.br (P.R.A.); 2 Department of Chemical, Materials and Industrial Production Engineering, University of Naples Federico II, Piazzale Tecchio, 80, 80125 Naples, Italy; roberto.teti@unina.it

**Keywords:** piezoelectric sensors, PZT, sensor monitoring, tool condition monitoring, electromechanical impedance, EMI, dressing, grinding process

## Abstract

Low-cost piezoelectric lead zirconate titanate (PZT) diaphragm transducers have attracted increasing attention as effective sensing devices, based on the electromechanical impedance (EMI) principle, for applications in many engineering sectors. Due to the considerable potential of PZT diaphragm transducers in terms of excellent electromechanical coupling properties, low implementation cost and wide-band frequency response, this technique provides a new alternative approach for tool condition monitoring in grinding processes competing with the conventional and expensive indirect sensor monitoring methods. This paper aims at assessing the structural changes caused by wear in single-point dressers during their lifetime, in order to ensure the reliable monitoring of the tool condition during dressing operations. Experimental dressing tests were conducted on aluminum oxide grinding wheels, which are highly relevant for industrial grinding processes. From the results obtained, it was verified that the dresser tip diamond material and the position of the PZT diaphragm transducer mounted on the dressing tool holder have a significant effect on the sensitivity of damage detection. This paper contributes to the realization of an effective monitoring system of dressing operations capable to avoid catastrophic tool failures as the proposed sensing approach can identify different stages of the dressing tool lifetime based on representative damage indices.

## 1. Introduction

In recent years, a relevant number of researches describe the application of tool condition monitoring (TCM) during material removal processes [1,2,3,4]. Studies on TCM consider grinding processes among the most vital material removal processes to produce parts requiring very high surface quality and geometrical accuracy in automotive, aerospace, biomedical, and other high-tech industrial sectors. During grinding, the wheel progressively loses its cutting capacity due wheel wear and fracture (attrition wear, grain fracture, bond fracture). Excessive grinding wheel wear leads to unexpected increase of power consumption, loss of workpiece surface quality, and occurrence of grinding burns. Dressing is then carried out to restore the abrasive wheel topography so that optimum performance can again be achieved [5]. According to Wegener et al. [6]: “grinding is dressing”. This slogan, kept in the grinding process community, points out the importance of dressing processes on the manufacturing results, besides all the other relevant parameters for grinding process effectiveness. In the dressing of conventional grinding wheels, single-point dressers made of natural or synthetic diamond are applied. Though very hard by nature, diamond also suffers wear during its use as a tool material. Dressers containing worn diamonds provide less sharpness on the grinding wheel and are consequently responsible for unsatisfactory grinding operations [4]. Hence, the development of monitoring systems for the process control during dressing is of utmost importance.

Researchers have made efforts to establish precision monitoring and diagnostic systems using direct and indirect tool condition monitoring techniques. Examples of direct monitoring for the tool wear measurement are the use of optical, laser and contact techniques following the approaches presented in [5,7,8] which requires that the machine be stopped for tool extraction and visual inspection, resulting in machine downtime and human user intervention cost. In contrast, indirect monitoring systems operate according to the variables that are prospectively effective for machining process monitoring, which can be measured by the application of appropriate physical sensors, such as: acoustic emission (AE), vibration, force, motor current and power. More sensors that are commonly used for online measurement are summarized in [9]. Furthermore, the signals measured by these sensors are subjected to digital signal processing and provide information for process optimization, i.e. the signal features are correlated with tool state and/or process conditions [9]. The use of sensor systems for tool condition monitoring in machining and grinding was already considered in the 1980s to enhance productivity, as reported by [10] (the status of research and industrial application between 1970s and 1995s was reported by these authors). Examples are: sensor monitoring of machining operations [11], tool wear monitoring in metal cutting [12], sensors for unmanned machining [13], and a review of the tool condition monitoring literature database [14]. Furthermore, numerous techniques and methods of signal processing for feature extraction on the basis of tool condition monitoring were reported in [9]. In addition, the evolvement of the tool condition monitoring techniques until nowadays were reported in many keynotes, case studies and review papers, such as: [1,2,9,15,16] (advanced topics in machining monitoring, innovative signal processing, sensor fusion and related applications were covered in these research works). As regard the dressing tool condition monitoring, there is a gap to fill, since the number of publications on this topic is still very small. Examples are: the detection of grinding wheel dressing time using force signals and wavelet transform signal processing [17], and the sensor fusion technology using AE signals combined with power signals [18]. In similar way, AE, vibration and power signal data were processed through artificial neural networks (ANN) and fuzzy systems to determine the dressing tool condition in terms of tool wear level classification and prediction [4,19,20,21]. Though findings on dressing process monitoring, as reported in the above cited papers, showed satisfactory classification rates and accuracy, considerable data processing time as well as high costs in terms of computing hardware were required. Therefore, it is worth to mention that, according [9] the luckily, today’s sensing devices are becoming increasingly dependable and low-priced and the signal processing capabilities of advanced algorithms and decision-making approaches are also rapidly progressing. This way, new and alternative techniques are constantly being proposed for machining and grinding process monitoring.

On the other hand, piezoelectric lead zirconate titanate (PZT) diaphragm transducers have emerged as a potential sensing devices for structural health monitoring (SHM) applications based on the electromechanical impedance (EMI) principle [22,23,24,25,26,27,28,29,30]. In this way, many researchers have been proposed approaches based on piezoelectric EMI technique in frequency-domain (by exciting and sensing a PZT bonded to the structure to be monitored a suitable frequency range) [28,31,32] or time-domain (i.e. the voltage responses of PZT can reflect the health status of the monitored structure) [33,34,35] for detection and evaluation of damages in structural members of civil and aerospace engineering (such as: concrete and aircraft members), composite members and many others. In addition, some other researchers proposed to use auxiliary techniques, such as artificial intelligence and smart interface to optimize the damage diagnosis based on the most sensitive frequency sub-range [36,37,38]. Furthermore, according to recent publications [39,40,41], this sensing technique proved to be highly promising for the monitoring of manufacturing process, with particular reference to grinding operations. PZT diaphragm transducers, such as conventional PZT ceramic or piezoelectric diaphragm sensors, are attached to or embedded in the host structure so as to provide for damage diagnosis: the PZT device acts simultaneously as sensor and actuator due to its direct and inverse piezoelectric effects [42]. In this research work, attention is given to the PZT diaphragm transducers, which are low-cost, compact and lightweight components widely used in a diversity of electronic devices (telephone ringers, audible alarms, piezoelectric transducers) [42]. In several case of studies, these PZT devices were used as sensors or actuators for various applications. As regards SHM applications, the use of PZT diaphragms transducers has successfully increased in aerospace and structural engineering for the detection of damages like cracks and corrosions in different structures such as concrete structures, metal parts of aircrafts and bridges, exploiting the EMI technique as sensing procedure [42,43,44,45]. According to these papers, the PZT diaphragm transducers presented damage indices with values similar to those of conventional PZT ceramic sensors, which are most commonly used from a commercial point of view. On the other hand, PZT diaphragm transducers were also used as passive transducers in partial discharge monitoring in power transformers [46,47], surface roughness discrimination [48], burn detection in grinding process [49].

In this paper, a new approach is proposed for dressing tool condition monitoring using low-cost PZT diaphragms based on the EMI technique. The main contribution of this paper is that the PZT diaphragm transducer along with the EMI technique provides a new alternative for dressing operation monitoring versus the conventional indirect TCM monitoring systems (such as those based on commercial sensors of force, vibration, and AE). In [44,46,50,51], the notable potential of a PZT diaphragm transducer was shown, due its excellent electromechanical coupling properties, simple feasibility, wide-band frequency response, and flexibility of use. In addition, it is worth mentioning their low cost compared to commercial sensors for force, vibration and AE detection: PZT diaphragm transducers have an average cost ranging from a few US dollar cents [52] versus the high average cost of the force, vibration and AE commercial sensors, ranging from hundreds to thousands of dollars [46]. Moreover, besides their low cost, PZT sensing devices based on the EMI technique use a simple methodology in terms of hardware instrumentation. In addition, the EMI technique possesses several advantages that have been confirmed in SHM applications, such as: (a) the ability to detect incipient damage, i.e. with the use of PZT transducers it is possible to detect changes in the high frequency structural dynamics at local scale which are directly associated with the presence of incipient damage [53]; (b) critical part inspections of structures usually require NDT methods, increasing the need of employment of this technique; and (c) this technique is a local damage detection method that is very effective for assessing the health of a nearby area [29]. These benefits are decidedly crucial for the monitoring of metal removal processes, such as grinding and dressing operations, particularly in industrial sectors where the pursuit of high-quality, low cost sensor monitoring techniques is becoming essential.

This work is an expansion of the research activities described in [40] where initial results, restricted to time-domain signal analysis, were presented. The scope of this paper is to illustrate a new broader approach for the monitoring of dressing tool structural condition during dressing operations, with particular reference to single-point dressing tools made of synthetic and natural diamonds. This new approach considers the real part of the impedance and employs a novel analysis based on damage metrics for feature extraction. Furthermore, at the present work a real application of EMI technique is considered which increase the possibility of its commercialization, since it is not been fully made yet and, regarding the SHM field, it has been conducted mainly under theoretical conditions. 

## 2. Electromechanical Impedance (EMI) Technique

### 2.1. PZT Diaphragm Transducers

According to [44], PZT diaphragm transducers are characterized by a very simple construction, consisting of a circular piezoelectric ceramic layer (active element) mounted on a circular metal plate (diaphragm). The ceramic layer is coated with a thin, metallic film operating as an electrode. Typically, the piezoelectric material is barium titanate or PZT (not pure but doped), and the metal diaphragm can be brass, nickel alloy or stainless steel.

According to IEEE-ANSI [54], the piezoelectric effect occurs in a material which, when subjected to a stress, produces a voltage output through the formation of an electric dipole in the material itself. The reverse effect also occurs, i.e. by applying an electric voltage to the opposite surfaces of the piezoelectric material a mechanical deformation is obtained. Thus, there is an interaction between the electrical and mechanical characteristics of the piezoelectric material. Based on these considerations, the direct piezoelectric effect (sensor) and reverse piezoelectric effect (actuator) in the transducer are given by Equations (1) and (2), respectively:

(1) $$D_{i}=d_{ikl}T_{kl}+\varepsilon_{ik}^{T}E_{k}$$

(2) $$S_{ij}=s_{ijkl}^{E}T_{kl}+d_{kij}E_{k}$$

where, $d_{ikl}$ and $d_{kij}$ are the piezoelectric constants, respectively; $E_{k}$ is the electric field, $D_{i}$ is the electrical displacement, $S_{ij}$ is the strain component, $\varepsilon_{ik}^{T}$ is the permittivity component at constant stress, $s_{ijkl}^{E}$ is the elastic compliance constant at constant electric field; $T_{kl}$ is the traction vector component, and the subscripts i, j, k, and l indicate the natural coordinate system of the piezoelectric crystal (values of 1, 2, 3) [42].

### 2.2. EMI Principle

According to [39], the EMI methodology is a type of non-destructive evaluation (NDE) technique that stands out for its simplicity and for using the previously mentioned PZT transducers (ceramics or diaphragms). These transducers are attached to or embedded in the host structure to be monitored and, due to the piezoelectric effect, a relationship is established between the mechanical properties of the structure and the electrical impedance of the transducer. This allows to monitor the variations of these properties by measuring the electrical impedance.

Thus, according to [36,45], the piezoelectric device must be excited over a suitable frequency range to obtain an electrical impedance ($Z_{E}\left(\omega \right)$) measurement (signature), which is related to the integrity of the monitored structure. Based on the piezoelectric constitutive Equations (1) and (2), the relation between electrical impedance of the piezoelectric transducer and mechanical impedance of the structure to be monitored can be determined by electromechanical models. According to [39,55,56], in one of the simplest models for a square PZT patch, the electrical impedance is given by Equation (3):

(3) $$Z_{E}\left(\omega \right)=\frac{1}{j\omega C_{0}}\|jZ_{T}{\left(\frac{s_{11}}{d_{31}\ell }\right)}^{2}\left[\frac{1}{2}\tan\left(\frac{k\ell }{2}\right)-\frac{1}{\sin\left(k\ell \right)}+\frac{Z_{s}}{j2Z_{T}}\right]$$

where $Z_{E}\left(\omega \right)$ is the electrical impedance, ω is the angular frequency, $C_{0}$ is the static capacitance for a square patch of size ℓ, k is the wave-number, $Z_{T}$ represents the mechanical impedance of the piezoelectric patch, $Z_{s}$ represents the mechanical impedance of the monitored structure, $d_{31}$, as mentioned previously, represents the piezoelectric constant, $s_{11}$ is the elastic compliance at constant electric field, ∥ indicates a parallel connection, and j is the unit imaginary number.

Several research works have proposed alternative measurement systems based on the EMI technique [28,29,57,58]. In this paper, attention is given to the EMI technique proposed in [59] to measure the electrical impedance through a multifunctional data acquisition (DAQ) device.

## 3. PZT Diaphragm Transducers Sensitivity Assessment

### 3.1. Pencil-Lead Break Method

The pencil-lead break (PLB) is a well-known method to characterize acoustic emission sensors [60]. Recently, the use of the PLB test to characterize PZT transducers, in particular low-cost PZT diaphragm transducers, has received increasing attention [61]. Almeida et al. [51] proposed the use of the PLB to assess the sensitivity of PZT diaphragm transducers for structural damage detection based on the EMI principle. The results conclusively demonstrate a very good match between the power spectral density (PSD) obtained by the PLB method and the damage indices computed using the electrical impedance signatures. Therefore, the PLB method can be considered a simple and effective tool to experimentally assess the sensitivity of piezoelectric transducers for damage detection by using the EMI technique.

The PLB is carried out by breaking a pencil lead on the structure or holder on which the transducer is mounted. The pencil-lead rupture releases an impulsive elastic wave that reaches the piezoelectric transducer which generates an impulsive voltage signal. This method is a reliable way of creating a wide band signal to analyze the propagation of waves in the investigated structure as well as the frequency response of PZT transducers. This method is a cheap and simple procedure to characterize an acoustic source and has been adopted as a standard (E976) [62]. In this paper, the PLB was applied to obtain the frequency response of the PZT diaphragm transducers utilized in the experiments.

### 3.2. Damage Indices

When the PZT diaphragm transducers are attached to the host structure, the electrical impedance displays peaks corresponding to the natural frequencies of the structure, and changes in these peaks are related to the onset of structural damages. Thus, damages can be identified by comparing the transducer electrical impedance when the structure is under healthy conditions (baseline) with the impedance measured after the structure has undergone an impairment. This comparison can be made by means of damage indices. One of the most employed indices in SHM is the Root Mean Square Deviation (RMSD) based on the Euclidean norm [51]. In this study, the RMSD index was calculated by Equation (4), as in [45,51]:

(4) $$RMSD=\overset{\omega_{F}}{\underset{k=\omega_{l}}{\sum }}\sqrt{\frac{{\left[Re(Z_{E,D}\left(k\right)-Re\left(Z_{E,H}\left(k\right)\right)\right]}^{2}}{Re^{2}\left(Z_{E,H}\left(k\right)\right)}}$$

where the subscripts H and *D* represent the healthy and damaged conditions, respectively, while $Re\left(Z_{E,H}\left(k\right)\right)$ and $Re\left(Z_{E,D}\left(k\right)\right)$ are the real part of the electrical impedance signatures of the structure under healthy and damaged conditions, respectively, measured at a frequency *k* ranging from $\omega_{1}$ (initial frequency) to $\omega_{F}$ (final frequency).

Another statistical damage index widely used in SHM is the Correlation Coefficient Deviation Metric (CCMD) based on the correlation coefficient between the two impedance signatures. In this study the CCDM index was calculated by Equation (5), as in [51].

(5) $$CCDM=1-C_{c}$$

where $C_{c}$ is the correlation coefficient, calculated using the real part of the electrical impedance signatures for the structure under healthy and damaged conditions in the same frequency range.

## 4. Material and Methods

### 4.1. Experimental Dressing Tests

Experimental dressing tests were carried out on a model RAPH 1055 surface grinding machine by Sulmecanica (Porto Alegre, Rio Grande do Sul, Brazil), equipped with a model 38A150L6VH aluminum oxide grinding wheel, with 355.6 mm × 25.4 mm × 127 mm dimensions, by Norton (Philadelphia, PA, USA). For comparison purposes, a first testing series with a single-point dresser made of synthetic diamond, obtained by chemical vapor deposition (CVD), and a second testing series with a single-point dresser made of natural diamond were performed to study the tool wear development. The experimental dressing tests were conducted in line with the procedures reported in [4,40]. Each test consisted in dressing passes throughout the grinding wheel cutting surface until the end of the dresser lifespan or the end of the grinding wheel lifespan. A dressing speed of 3.45 mm/s and a dressing depth of 40 µm were kept constant during all the tests. The overlap ratio, *U_d_*, was set equal to 1 at the beginning of the testing. Both testing series were carried out without cutting fluid in order to cause a faster wear development.

Due to the presence of material impurities and the specific methods to produce the diamonds, the number of passes for the CVD diamond dressing tool differed from the number of passes for the natural diamond dressing tool. It was verified that the CVD diamond presented a higher resistance to wear due to its manufacturing method and it was not possible to reach the end of its lifespan within the lifespan of the grinding wheel. That is, after 600 dressing passes, it became impracticable to continue the test because the grinding wheel diameter was much reduced and out of safe operation (end of grinding wheel lifespan). On the other hand, the natural diamond reached the end of its lifespan at 300 dressing passes.

**Figure 1:**
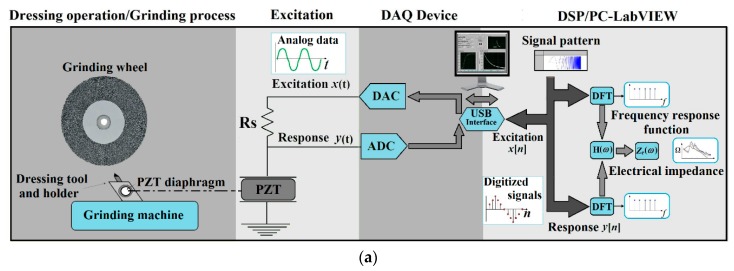

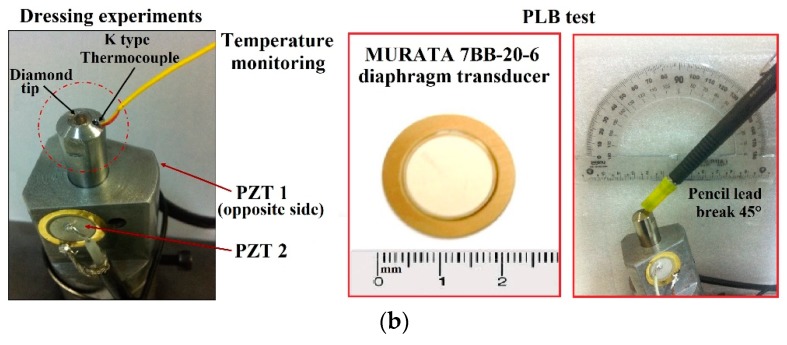
Proposed sensing setup for dressing tool condition monitoring: (**a**) EMI technique-based system; and (**b**) dressing experiments and PLB setup.

To perform wear measurements of the dresser tip, model DIGIMICRO 2.0 digital microscope, by DNT (Fremont, CA, USA), was used, with magnifications from 10× to 200×, equipped with software to measure distances and areas. The magnification adopted was 20× and the wear area was measured from the side position of the diamond tips. Figure 1 presents a scheme of the test bench.

### 4.2. DAQ Device and Sensing System

The EMI technique for impedance measurement [59] was utilized as shown in Figure 1a. Low-cost PZT diaphragm transducers, model 7BB-20-6, manufactured by MURATA Inc. (Sao Paulo, Brazil) [52], with brass plate diameter of 20 mm, were used as transducers in the experimental tests. As shown in Figure 1b, two diaphragms, PZT1 and PZT2, were bonded on the dresser holder at opposite equidistant positions in order to ensure a proper comparison for the different transducer locations. 

According to Figure 1a, for each mounted transducer the DAQ device should have at least one analog output to provide the excitation signal x(t) through the digital-to-analog converter (DAC) and one analog input for the acquisition of the signal response y(t) from the transducer through the analog-to-digital converter (ADC). Signals x[n] and y[n] are the digital forms of the transducer excitation and response signals, respectively, to be subjected to signal processing procedures on PC. The DAQ device and the personal computer (PC) are connected by a universal serial bus (USB). The transducer is connected to the DAQ device through a resistance Rs [39]. A National Instruments DAQ device, model NI USB-6221, was used to measure the electrical impedance of the PZT diaphragms transducer at sampling rate 250 kS/s. The PZT diaphragm transducers were excited through a 2.2 kΩ resistor (RS in Figure 1a) by a chirp signal of magnitude 1 V and frequency from 0 to 120 kHz. The mean value of three measurement repetitions was calculated to ensure accuracy. A computer equipped with LabView software code was used to record the impedance signatures from the DAQ device.

The real part of the impedance signal was considered for analysis and the RMSD and CCDM indices were calculated by Equations (4) and (5), respectively, for several frequency sub-ranges between 0 and 120 kHz. The impedance measurements were performed at dressing pass intervals, variable with the test conditions, with the scope to detect variations in the impedance signal for different damage levels. Three damage conditions were defined: healthy diamonds (initial tool condition with no damage) at the beginning of the test; damage 1 (intermediate tool damage condition) at the middle of the test duration; damage 2 (final tool damage condition) at the end of the dressing test, i.e. when the dresser or grinding wheel reached their end of life. For the CVD diamond dressing tool, intermediate and final tool damage conditions were considered at dressing passes # 300 and # 600, respectively. For the natural diamond dressing tool, intermediate and final tool damage conditions were considered at dressing passes # 150 and # 300, respectively.

### 4.3. PLB Method and Temperature Monitoring

The PLB was performed, according to [51] and the E976 standard [62], by using a mechanical pencil equipped with lead type 2H of 3 mm length and 0.5 mm diameter. The lead break was obtained manually, with 45° angle between structure and lead, at a distance of 50 mm from the transducer (Figure 1 b). The lead was broken on each dresser diamond tip in order to compare the response of both PZT1 and PZT2 diaphragm transducers for both CVD and natural diamond dressing tools. The output voltage signals were detected at sampling frequency 2 MS/s by a DL850 oscilloscope (Yokogawa, Sao Paulo, Brazil). An average value vector of the raw response signal was obtained from 10 PLB repetitions for each PZT diaphragm transducer. The analysis was performed in the frequency domain calculating the power spectral density (PSD) [51] using the Welch method with a Hanning window of 256 values and an overlap of 50%.

Changes in temperature are known to provide variations in the electrical impedance as well as in the damage indices RMSD and CCDM. Consequently, this kind of influence can issue a false positive diagnosis [56]. Therefore, the impedance measurements and the PLB were carried out at constant temperature of 30 °C, monitored using a model MT455 digital thermometer by MINIPA (Houston, TX, USA), equipped with a K type thermocouple. To fix the thermocouple, a hole was made in the dresser body (Figure 1 b) near the diamond, where the thermal conductivity is high, i.e., 1000 W/(mK) (in steel it is 50.2 W/(mK)) [5]. Although the dressing tests were performed without coolant to induce wear damage more rapidly, a cooling system was adopted for the host structure of the PZT diaphragm transducers, ensuring that the transducers were protected against any thermal damage.

## 5. Results and Discussion

### 5.1. Tool Wear Analysis

**Figure 2:**
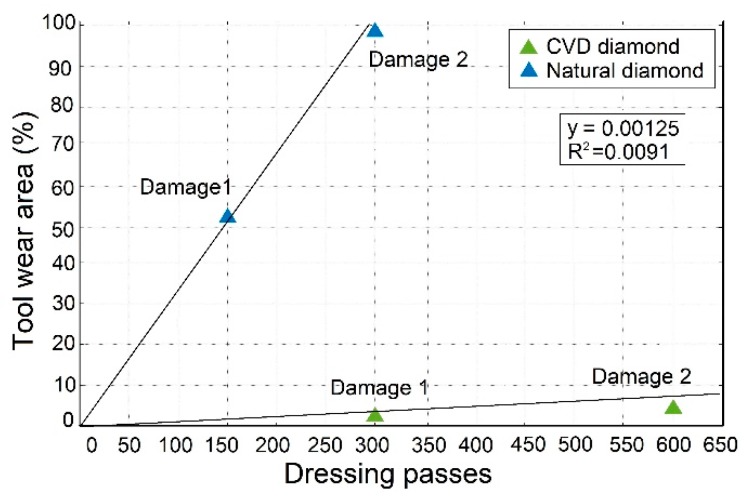
Diamond dressing tools wear analysis.

To validate the effectiveness of the proposed approach, a tool wear analysis was performed. Figure 2 reports the worn area, evaluated as a percentage, for both CVD and natural diamond dressing tools versus number of dressing passes. This analysis aims at confirming whether the proposed sensor monitoring approach using low-cost PZT diaphragm transducers and EMI method are able to detect changes in the dressing tool conditions. For each dressing test, the tool wear conditions were categorized at different dressing passes, as shown in Figure 2. It can be observed that the condition of the CVD diamond did not change significantly with increasing number of dressing passes, which demonstrates its high resistance to wear. On the other hand, a significant difference can be observed for the natural diamond, which displayed a much lower resistance to wear. The different behavior of the CVD and natural diamond tools are in agreement with [4,63,64,65], which means that the diamond material has a strong influence on the tool lifetime and its wear development.

Furthermore, it can be clearly noted in Figure 2 that both dressing tool diamond tips presented the same tendency during the dressing experiments, i.e. the worn area and the number of dressing passes increases linearly. However, the CVD diamond presented less than 10% of worn area at 600 passes, and the natural diamond, in contrast, presented 100% of worn area already at 300 dressing passes.

### 5.2. PZT Diaphragm Transducer Characterization

**Figure 3:**
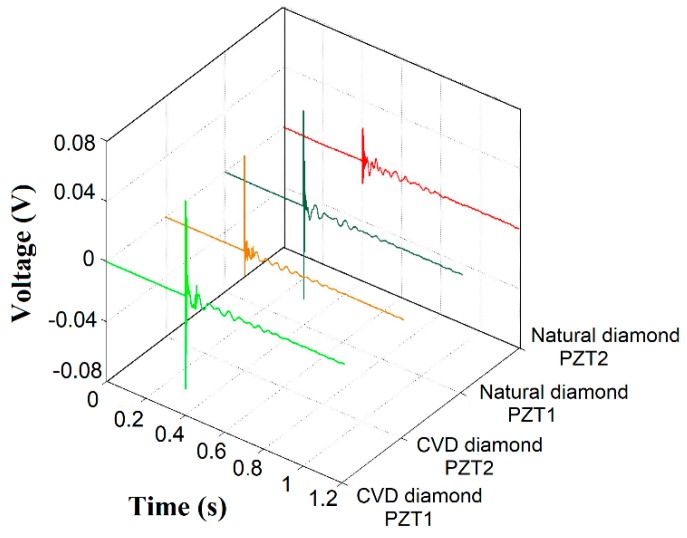
PLB results: Voltage signals.

**Figure 4:**
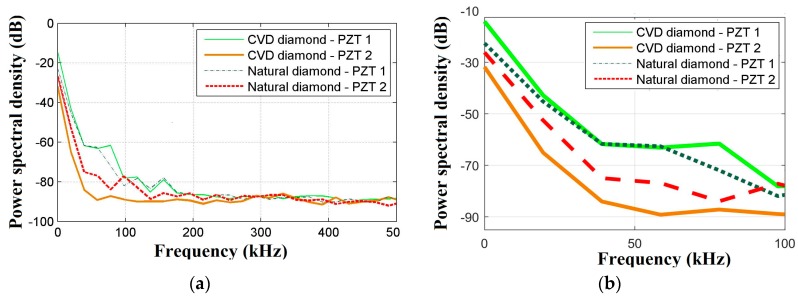
PLB test results: (**a**) frequency response analysis via PSD; and (**b**) enlarged view into the range of 0–100 kHz.

Figure 3 reports a plot of voltage signals in the time domain, for both the PZT1 and PZT2 diaphragms, detected during the PLB for both CVD and natural diamond dressing tools. The signals from PZT1 were similar in terms of voltage levels for both CVD and natural diamond dressing tools. In contrast, the signals from PZT2 presented a lower magnitude under the same conditions, which can be attributed to some minor distance differences between the two transducers and the PLB location due to asymmetric testing conditions. In addition, PZT characteristics, such as the capacitance, can vary up to 20%, according to [42,44,45]. The power spectral density (PSD) of these signals was computed, as reported in Figure 4a,b. As expected, the same magnitude behavior as in the time domain is observed also in the frequency domain for both PZT1 and PZT2 diaphragm transducers. However, according to the enlarged view into the range of 0–100 kHz shown in Figure 4b, magnitude differences can be better verified in the frequency domain, where a significant difference of up to 30 dB in CVD diamond dressing tool was observed for the PZT1 (set at approximately at −60 dB) and PZT2 (set at approximately at −90 dB) diaphragm transducers. Also, the diamond type influenced the PZT diaphragm transducer frequency response as shown by the fact that the magnitude differences between PZT1 and PZT2 diaphragm transducer are much smaller for the natural diamond. Moreover, it can be clearly noted that, in general, the transducers display more sensitivity in terms of damage detection for frequencies below 200 kHz.

### 5.3. Dressing Tool Condition Monitoring Though Impedance-Based Sensors

**Figure 5:**
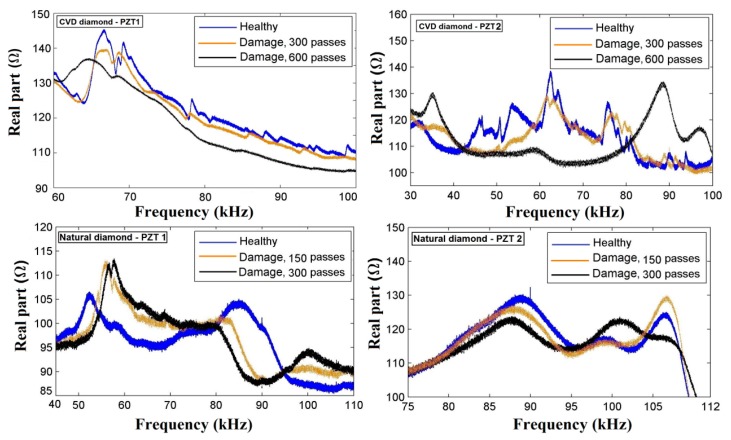
Impedance signatures for both PZT1 and PZT2 diaphragms obtained for the diamond-dressing tools under the healthy and damaged conditions.

Figure 5 shows a comparison of the impedance signatures obtained for the diamond dressing tools under healthy and damaged conditions, respectively. It is worth mentioning that the different behaviors of the CVD and natural diamond tips in terms of mechanical properties, as described in [41,42], can also contribute to the different behaviors of the impedance signatures for both PZT1 and PZT2 diaphragm transducer, as shown in Figure 5. Furthermore, because impedance signatures differ, each transducer has a specific frequency range that is more relevant to analyze. To support this claim, it is possible to find examples in the literature [39,45]. In [45], a comparative analysis of the effects of three transducer mounting methods on the sensitivity for structural damage detection based on conventional EMI and transfer frequency response function (FRF) methods was presented. The experimental results indicated that the different transducer mounting procedures influenced the damage detection capability as well as the frequency bands most sensitive to damage. In [14], a new approach for the monitoring of surface grinding using the EMI method was proposed. PZT diaphragm transducer were mounted on different structural components, such as workpiece or holder, and the results indicated that each transducer had a specific frequency range which was more appropriate to calculate the RMSD and CCDM damage indices. 

Thus, only the specific frequency ranges that were more sensitive to damage are shown in Figure 5 to ensure a proper comparison: the reference to the specific frequency ranges allows analyzing the impact of each transducer mounting location (PZT1 and PZT2) as well as of each tool type (CVD and natural diamond dressing tools) on the sensitivity of the monitoring approach for dressing tool damage condition identification. Figure 5 shows that impedance signatures are significantly different for diverse experimental conditions. As regards the impedance signatures of the PZT1 diaphragm transducer and the CVD diamond dressing tool, a narrow frequency band from 60 kHz to 125 kHz was selected. It can be observed that, within this range, the most significant frequencies capable to describe damage conditions, (impedance signatures at dressing pass # 300 and # 600, respectively) were between 65 kHz and 70 kHz. In contrast, as regards the impedance signatures of the PZT1 diaphragm transducer and the natural diamond dressing tool, the narrow frequency band from 40 kHz to 110 kHz was considered. In this case, the impedance signatures presented higher magnitudes as dressing tool damages occurs (at dressing pass # 150 and # 300, respectively). In this frequency band, resonance peaks become predominant between 55 kHz and 60 kHz due to the tool damage when compared with the resonance peaks related to the undamaged structure (baseline).

As regards the impedance signatures of the PZT2 diaphragm transducer and the CVD diamond dressing tool, the narrow frequency range from 30 kHz to 100 kHz was selected (Figure 5). A similar behavior can be noted as for the PZT1 diaphragm transducer and the same CVD diamond dressing tool in terms of magnitude of the impedance signatures. This means that the impedance signatures had lower magnitudes in the case of tool damage. Moreover, predominant resonance peaks in this frequency range were observed at 60 kHz corresponding to the impedance signatures of the healthy tool and at dressing pass # 300, respectively, as well as at 35 kHz and 90 kHz corresponding to the impedance signatures at dressing pass # 600. Finally, as regards the impedance signatures of the PZT2 diaphragm and the natural diamond dressing tool, a narrow frequency band from 75 kHz to 112 kHz was selected. In this case, it was possible to confirm some frequencies that were most sensitive to damage inside of this selected range, such as 88 kHz and 107 kHz corresponding to damages at 150 passes and 300 passes, respectively.

**Figure 6:**
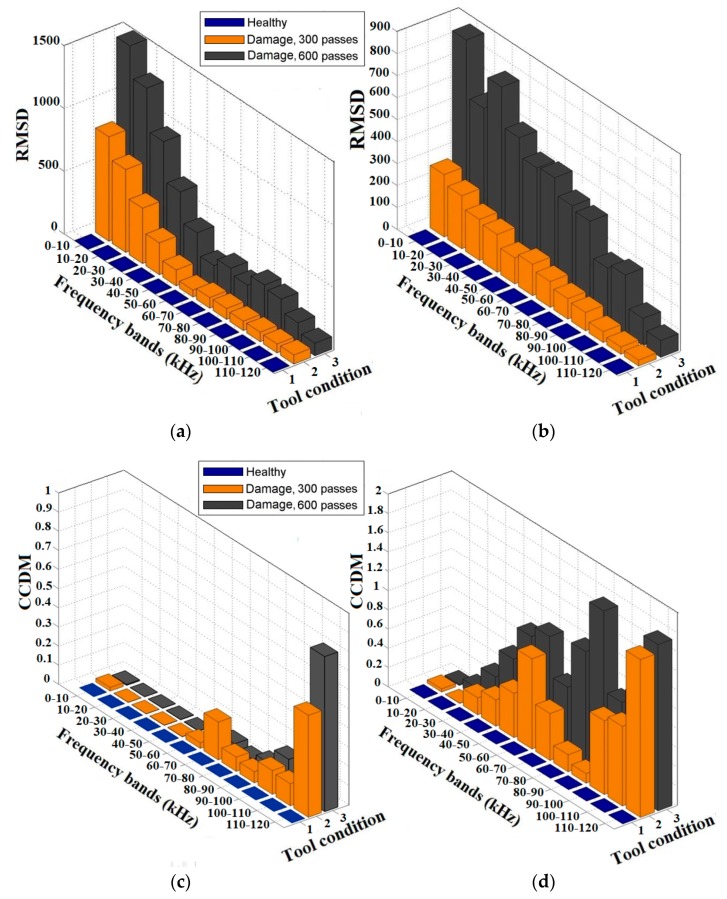
Damage indices results of the CVD diamond-dressing tool: (**a**) RMSD of PZT1; (**b**) RMSD of PZT2; (**c**) CCDM of PZT1; and (**d**) CCDM of PZT2.

Thus, according to results presented in Figure 5, the proposed sensor monitoring approach was able to detect structural damage in dressing tools, for all the considered test conditions, since considerable impedance signature variations can be observed between healthy and damaged tool conditions. The effectiveness of the proposed sensor monitoring approach for tool damage detection can be additionally evaluated through an analysis based on the RMSD and CCDM damage indices, calculated for the frequency ranges displaying the highest sensitivity to tool damage [45]. However, the selection of the most appropriate frequency ranges for the RMSD and CCDM indices calculation needs to be carried out through analytical approaches or artificial intelligence paradigms [36,37,38]. For this reason, in this paper a wide frequency band of 0–120 kHz was considered and subdivided into single sub-ranges equal to 10 kHz for a more thorough evaluation of damage indices [42,45]. The calculation considered the healthy tool state as baseline, corresponding to the diamond dressing tool condition before experimental dressing tests. The results obtained with the RMSD and CCDM indices in the case of CVD diamond dressing tool are shown in Figure 6.

The RMSD index values for the PZT1 diaphragm transducer are shown in Figure 6a, and those for the PZT2 diaphragm transducer in Figure 6b. The higher values of the RMSD index are primarily concentrated at low frequencies for both PZT1 and PZT2 diaphragm transducer. Hence, the RMSD index presented a decreasing tendency in terms of magnitude for high frequency sub-ranges, especially at 100–110 kHz and 110–120 kHz, respectively. Such behavior was also predicted by PSD from PLB results (Figure 4). This result is in agreement with [45] where higher values of the RMSD index were mainly concentrated at low frequencies when different transducer mounting methods were considered. Moreover, the RMSD index values for the PZT 1 diaphragm transducer presented higher magnitudes compared with those for the PZT2 diaphragm transducer. It can be noted that the RMSD index increases as dressing tool damage occurs, since the values for the damage condition at 600 dressing passes are higher than those for the damage condition at 300 dressing passes, in agreement with [39]. Figure 6c,d present the results of the CCDM index for both PZT 1 and PZT 2 diaphragm transducer. The tendency is opposite to the one of the RMSD index. Higher values of the CCDM index are primarily concentrated at high frequencies for both PZT 1 and PZT2 diaphragm transducer, in particular for the PZT2 diaphragm transducer (Figure 6d) where the values of the CCDM index were significantly higher than those of the PZT 1 diaphragm.

**Figure 7:**
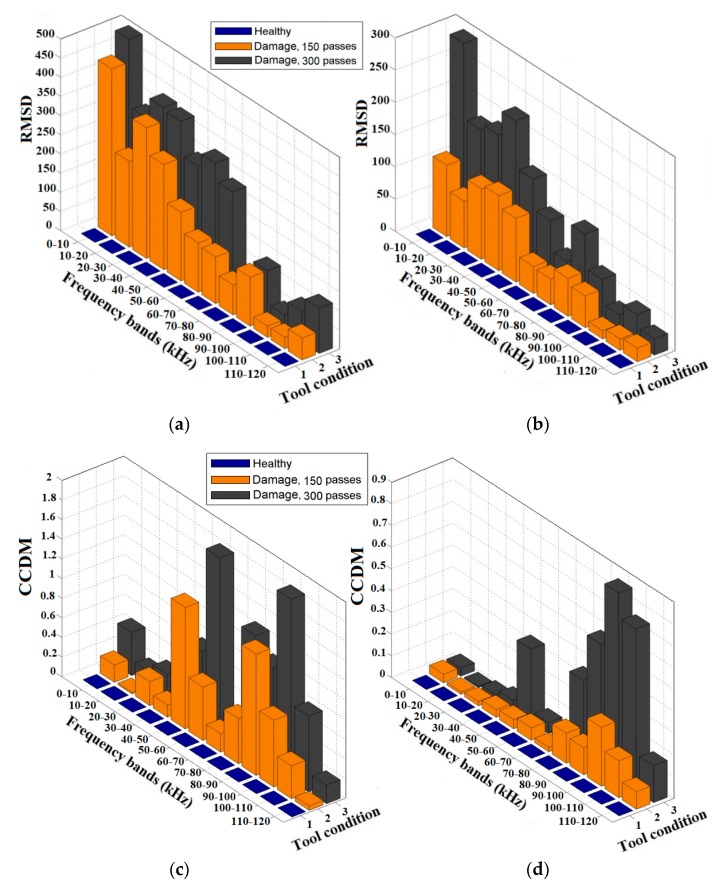
Damage indices results of the natural diamond-dressing tool: (**a**) RMSD of PZT1; (**b**) RMSD of PZT2; (**c**) CCDM of PZT1; and (**d**) CCDM of PZT2.

The results obtained with the RMSD and CCDM indices for the natural diamond dressing tool are shown in Figure 7. The RMSD index values for the PZT1 diaphragm transducer are shown in Figure 7a, and those for the PZT2 diaphragm transducer in Figure 7b. In the case of the natural diamond, the results based on the RMSD index are very similar to those of the CVD diamond (Figure 6). Again, the higher index values are mainly concentrated at low frequencies and decrease going towards high frequencies. However, the differences are related to the magnitude, since the RMSD index values for the natural diamond dressing tool have relatively lower magnitudes than those obtained for the CVD diamond, similarly to [39].

Figure 7c,d presents the CCDM index values of the natural diamond dressing tool for both PZT1 and PZT 2 diaphragm transducers. The CCDM index values of the natural diamond, in contrast with the RMSD index, present a very different behavior from those of the CVD diamond (Figure 6). The CCDM index values also behave differently for the PZT1 and the PZT2 diaphragm transducers. The results for the PZT1 diaphragm transducer (Figure 7c) did not show any tendency because of the irregular magnitude variations observed for all frequency sub-ranges. On the other hand, it is worth mentioning that magnitude variations can be also related to the capacitive reactance of the PZT diaphragm transducer (1/$j\omega C_{0}$, as shown in (1)), which has direct effects on impedance signatures. However, these effects should be less significant, especially at the frequency ranges considered in this paper where the capacitive reactance is low, according to [42,45]. Therefore, it is possible to see an agreement with the literature [39,41]: the CCDM index increases as dressing tool damage occurs, since again the values for the damage condition at 600 dressing passes are higher than those for the damage condition at 300 dressing passes for all frequency bands.

Finally, as regards the results of the CCDM index for the PZT2 diaphragm transducer (Figure 7d), they presented a milder behavior and were more susceptible to dressing tool damage detection than the PZT1 diaphragm transducer. Again, the high index values are primarily concentrated at high frequencies, especially those for the damage condition at 300 dressing passes where the CCDM values are significant higher at high frequency. 

Thus, the results reported in Figure 6 and Figure 7 indicate that the RMSD index is very sensitive to damage development in diamond dressing tools based on the proposed approach, for both piezoelectric diaphragm transducers (PZT1 and PZT2) and for both structure types (CVD and natural diamond tips) under the experimental conditions of this paper. The CCDM index has an intermediate sensitivity that significantly depends on the structure type and the transducer mounting location, as observed from their different behavior in comparison with the RMSD index. However, the results show that the transducer mounting location and the dressing tool diamond type influence directly the magnitude and frequency bands of the damage indices.

Table 1 summarizes the main characteristics that influenced the experimental results by reporting the maximum values of the considered variables, i.e. the maximum values obtained with PSD from PLB test, the real part of the impedance signatures, the RMSD and CCDM index values.

The values of Damage 1 are related to 300 dressing passes for the CVD diamond dressing tool and to 150 dressing passes for the natural diamond dressing tool, respectively. On the other hand, the values of Damage 2 are related to 600 dressing passes for the CVD diamond dressing tool and to 300 dressing passes for the natural diamond dressing tool. Table 1 clearly shows that the signals from PZT1 for the CVD diamond dressing tool present higher magnitude values of PSD as well as higher magnitude values of the impedance signatures for the RMSD index. However, for the CCDM index, the highest value was obtained for the impedance signature of the PZT1 corresponding to the natural diamond dressing tool. In any case, it can be observed that RMSD and CCDM indices undergo considerable variations as dressing tool damage takes place. In addition, the values obtained for both RMSD and CCDM indices corresponding to Damage 2 are higher than those corresponding to Damage 1. Therefore, it is possible to prevent the dressing tools to suffer the total collapse of their diamond tips if the dressing process is stopped when the values of Damage 1 are reached. To this end, it is necessary to calculate the RMSD and CCDM indices through a real-time tool condition monitoring system based on the EMI method. Then, using a threshold previously set in relation to the variations of magnitude and frequency of the impedance signatures, it is possible to avoid that the dressing operation be performed with damaged dressers.

## 6. Conclusions

In this paper, a new approach for sensor monitoring of single-point diamond dressing tool conditions by means of PZT diaphragm transducers and EMI technique was presented. Experimental dressing tests showed that the proposed sensing method is capable to effectively detect structural damage in the relatively small diamond tool tips, which is a decided improvement in comparison with conventional SHM applications. The results were in good agreement with those observed in other research works cited herein, showing that the impedance signatures become more predominant with the increase of both RMSD and CCDM damage indices as the dressing conditions become more severe. The type of monitored dressing tools in terms of tool material (CVD and natural diamond tips) as well as the mounting location of the PZT1 and PZT2 diaphragm transducers influenced directly the damage detection capability of the proposed sensing methodology. By using the proposed sensing methodology, the dressing operation can be optimized to prevent operation with worn or damaged dressers, avoid catastrophic tool failures, ensure high quality and precision of the ground parts, and provide significant benefits to manufacturing chains based on grinding processes.

## Figures and Tables

**Table 1:** Main features obtained from maximum values for each analysis in dressing tests.

PZT Diaphragm Transducer and Dressing Tool	PSD (dB) from PLB Test	Impedance Signatures from Real Part (Ω)			RMSD Indices		CCDM Indices	
		Healthy	Damage 1	Damage 2	Damage 1	Damage 2	Damage 1	Damage 2
PZT1, CVD diamond	−14.23	140.4	134.1	132.1	831.3	1490	0.534	0.815
PZT2, CVD diamond	−31.85	138.5	129.5	134.7	284.1	857.6	1.643	1.731
PZT1, natural diamond	−22.66	106.6	113.2	113.4	435.2	495	1.236	1.845
PZT2, natural diamond	−26.23	132.4	129.9	124	116.6	289.4	0.271	0.860
